# Emerging pharmaceutical therapeutics and delivery technologies for osteoarthritis therapy

**DOI:** 10.3389/fphar.2022.945876

**Published:** 2022-11-17

**Authors:** Cheng-Yu Shentu, Ge Yan, Dong-Chen Xu, Yong Chen, Li-Hua Peng

**Affiliations:** ^1^ College of Pharmaceutical Sciences, Zhejiang University, Hangzhou, China; ^2^ State Key Laboratory of Quality Research in Chinese Medicine, Macau University of Science and Technology, Macau, Macau SAR, China

**Keywords:** osteoarthritis, pathological mechanism, drug candidates, delivery technologies, treatment strategies

## Abstract

Osteoarthritis (OA) is one of the most common joint degenerative diseases in the world. At present, the management of OA depends on the lifestyle modification and joint replacement surgery, with the lifespan of prosthesis quite limited yet. Effective drug treatment of OA is essential. However, the current drugs, such as the non-steroidal anti-inflammatory drugs and acetaminophen, as well as glucosamine, chondroitin sulfate, hyaluronic acid, are accompanied by obvious side effects, with the therapeutic efficacy to be enhanced. Recently, novel reagents such as IL-1 antagonists and nerve growth factor inhibitors have entered clinical trials. Moreover, increasing evidence demonstrated that active ingredients of natural plants have great potential for treating OA. Meanwhile, the use of novel drug delivery strategies may overcome the shortcomings of conventional preparations and enhance the bioavailability of drugs, as well as decrease the side effects significantly. This review therefore summarizes the pathological mechanisms, management strategies, and research progress in the drug molecules including the newly identified active ingredient derived from medicinal plants for OA therapy, with the drug delivery technologies also summarized, with the expectation to provide the summary and outlook for developing the next generation of drugs and preparations for OA therapy.

## Introduction

There are more than 400 million patients affected by osteoarthritis (OA) worldwide, with a global total prevalence of OA of 15%, causing significant economic burdens (Hiligsmann et al., 2013). OA is resulted from a combination of risk factors such as age, obesity, knee malalignment, biomechanical loading of joints, low-grade systemic inflammation, etc. At present, non-pharmacological approaches and surgical therapies are the most commonly used treatments for OA. Pharmaceutical treatments recommended by international guidelines to treat OA can merely alleviate symptoms or have obvious side effects if long-term use (Robinson et al., 2018; Ferguson et al., 2018; van de Graaf et al., 2018; Ferreira et al., 2019). Therefore, it is urgent to develop novel therapeutics to treat OA. In the past years, more and more therapeutic molecules, including many natural compounds isolated from medicinal plants, as well as multiple new delivery technologies to increase the absorption and decrease the side effects of the therapeutic candidates have been investigated This review summarized the progress of these emerging pharmaceutical therapeutics and the delivery technologies, with the therapeutic effects of active molecules extracted from traditional Chinese medicine are highlighted, with the expectation to identify much more alternatives and new options for the treatment of OA.

## Pathophysiology of osteoarthritis

**FIGURE 1:**
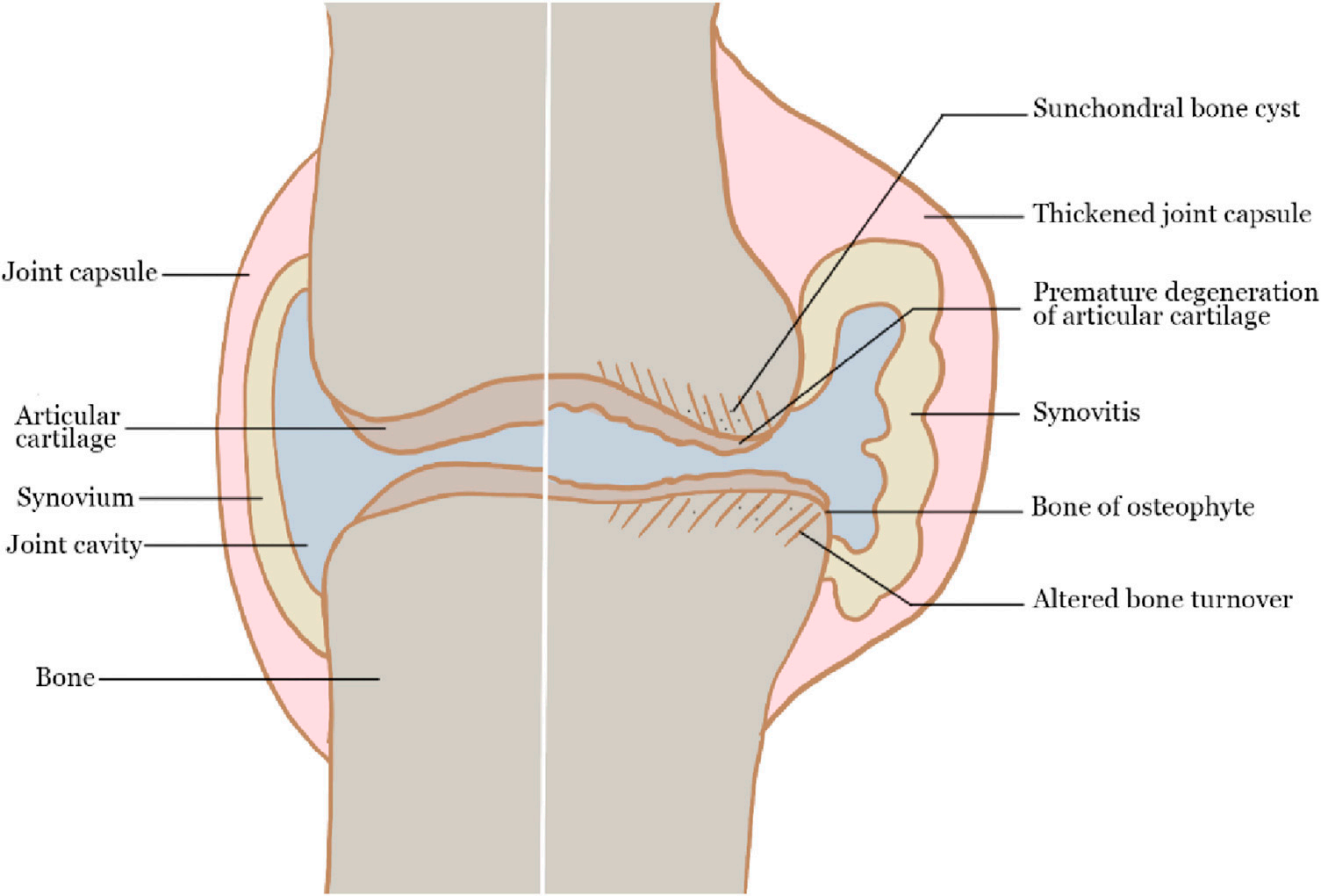
The pathophysiology of OA.

OA is a whole joint disease that affects the hyaline articular cartilage, subchondral bone, ligaments, capsule, synovium and periarticular muscles (Martel-Pelletier et al., 2016). The integrity of cartilage structure is damaged during the OA process, making cartilage more vulnerable to external stimulation (Loeser et al., 2016; Hunter and Bierma-Zeinstra, 2019). At the initial stage, articular cartilage was degraded from the cartilage surface and cracks emerged gradually, then enlarged into the cartilage calcification area. The degradation products and pro-inflammatory mediators released during this process induced synovial hyperplasia and inflammatory response in the surrounding synovium, as well as vascular infiltration in the subchondral bone. Although hypertrophic chondrocytes began to proliferate in an attempt to repair cartilage, the self-repair ability of articular cartilage is limited, resulting in the development of OA, expressed as the changes in the structure and properties of articular cartilage. The scheme of the main pathophysiology of OA is shown in Figure 1.

**FIGURE 2:**
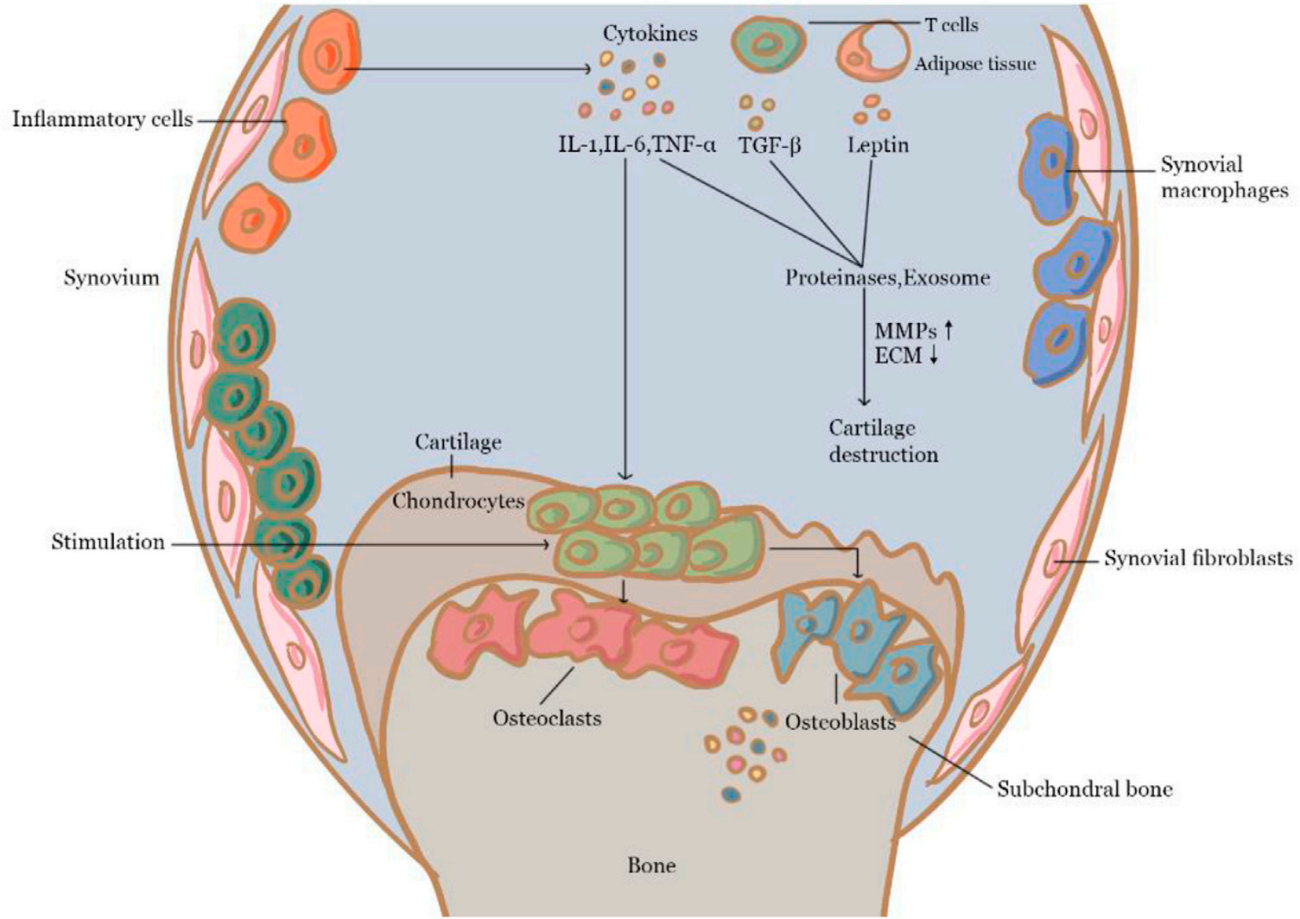
Cytokines and mechanism of OA.

Inflammation is thought to play an important role in the formation and progression of OA. There is a clear link between the progression of cartilage degeneration and the existence of reactive or inflammatory synovium (Felson, 2006). For example, inflammatory substances like proinflammatory cytokines are important mediators of the altered metabolism and increased catabolism of joint tissue in OA (Ayral et al., 2005; Pozgan et al., 2010). Among them, IL-1β, TNF, and IL-6 are several main proinflammatory cytokines involved. Particularly, IL-1 has been found to accelerate cartilage degradation and nociceptive pathway stimulation by activating the nuclear factor kappa-B (NF-ĸB) pathway (Benito et al., 2005). IL-1 is produced by chondrocytes and synovial nucleated cells in patients with OA, and IL-1 stimulated Matrix metalloproteinase (MMP)-1 and MMP-13 expression in chondrocytes (Mengshol et al., 2000). TNF-α has been found to be elevated in the synovial fluid, synovial membrane, subchondral bone, and cartilage of OA patients in various studies (Stannus et al., 2010; Xue et al., 2013). In-articular chondrocytes, TNF-α upregulates MMP-13 and stimulates the production of iNOS, cyclooxygenase (COX)-2, IL-6, and PGE2 (Guerne et al., 1990, Séguin and Bernier, 2003; El Mansouri et al., 2011), while suppressing the synthesis of type II collagen, proteoglycans, and proteoglycan-binding proteins (Xue et al., 2013). TNF-α blocks mesenchymal stem cells (MSCs) differentiation into osteoblasts by Notch activation (Zhao, 2017) and can regulate bone remodeling. IL-6 also associates with joint tissue hyperalgesia and hypersensitivity, as well as cartilage degeneration (Brenn et al., 2007) such as by inhibiting type II collagen formation while increasing the production of MMPs (Rowan et al., 2001; Porée et al., 2008). The IL-6-activated JAK/STAT and mitogen-activated protein kinase (MAPK) pathways have been found to upregulate the level of MMP-1, MMP-3, and MMP-13 in human chondrocytes (Aida et al., 2012). IL-6 also has been discovered to have a key function in mediating effects on the subchondral bone layer (Chenoufi et al., 2001; Kwan Tat et al., 2004; Sakao et al., 2009). TGF-β was demonstrated to be able to suppress hypertrophy in MSCs and articular chondrocytes (Yang et al., 2001; Zhang et al., 2004). TGF-β binds to heterotypic TGF receptors in chondrocytes, causing the phosphorylation of the heteromeric SMAD2/3/4 complex. The active small molecules against decapentaplegic (SMAD) complex subsequently translocates to the nucleus, where it interacts with co-transcription factors to control gene transcription, including chondrogenesis stimulation and degeneration and mineralization suppression (Harradine and Akhurst, 2006; Chen et al., 2012; Shen et al., 2013). Leptin is primarily produced by adipocytes, and it can help chondrocytes in the growth plate proliferate and differentiate (Wang et al., 2012). Through the JAK/STAT and MAPK pathways, it can stimulate chondrocytes (Ben-Eliezer et al., 2007) and is essential for chondrocyte hypertrophy (Kishida et al., 2005; Simopoulou et al., 2007). During OA, leptin can also upregulate the expression of MMPs, resulting in cartilage degradation (Toussirot et al., 2007; Vuolteenaho et al., 2009; Koskinen et al., 2011). Leptin binds to hypothalamic receptors and activates osteoblasts *via* β2 adrenergic receptors in the central route (Takeda et al., 2002). It can inhibit osteoblast proliferation, and boost bone resorption of osteoclasts (Fu et al., 2005). Within the peripheral pathway, leptin binds to human MSCs, promoting proliferation and differentiation into osteoblasts (Astudillo et al., 2008). In the past several years, exosomes were shown to be able to facilitate cell-to-cell communication and control a variety of biological processes such as immune response and inflammation (Salvi et al., 2018; Console et al., 2019). Because of its direct and close contact with the synovial membrane, articular cartilage, and other types of joint tissue, exosomes contained in the synovial fluid is valuable for monitoring pathological changes in the joint area (Kolhe et al., 2017). However, relevant research is still in its early stages, and the molecular mechanism of exosome-receptor cell contact remains further investigation. Cytokines and mechanisms related to OA are shown in Figure 2.

### Treatment strategies

Currently, OA is likely to be treated with lifestyle modification, pharmaceutical drugs, surgery, and also in combination. In the early stage of OA, lifestyle modifications such as swimming or Tai chi show to help patients with OA to alleviate pain and recover functions (Vignon et al., 2006). Orthotics have been shown to alleviate discomfort and increase function (Hussain et al., 2016). Besides these physical treatments, acetaminophen and non-steroidal anti-inflammatory drugs are widely used to resist inflammatory reactions and relieve pain, glucocorticoids, hyaluronic acid, chondroitin sulfate and glucosamine are also frequently used. Oral NSAIDs are the mainstay of the pharmacologic management of OA at present. NSAIDs can exert anti-inflammatory effects (Liu et al., 2013; Chen et al., 2017; Zhang et al., 2020a), and have been demonstrated to improve central and peripheral sensory thresholds and lessen pain. NSAIDs can diminish hyperalgesia by reducing the inflammatory and cellular immunological reactions generated by inflammatory mediators (Argoff, 2011). Furthermore, researchers discovered that NSAIDs preserve cartilage by efficiently inhibiting the damage caused by local inflammatory reactions to chondrocytes and synovial cells (Gunson et al., 2012). All of these drugs, however, have the potential for gastrointestinal, hepatic, and cardiorenal side effects, which increase with dose and treatment duration (Bjarnason, 2013; Ghosh et al., 2015; García-Rayado et al., 2018; Tai and McAlindon, 2018; Bindu et al., 2020). Chondroitin sulfate (CS) is a natural glycosaminoglycan present in cartilage and the ECM. In chondrocytes and synovial membranes, CS inhibits NF-κB activation and nuclear translocation (Uwe, 2008). To treat OA, CS is frequently combined with glucosamine (Bishnoi et al., 2016). Its capacity to relieve symptoms or reduce the structural progression of OA has been studied in clinical trials. The results have been contradictory, owing to variances in patient demographics and CS purity (Uebelhart et al., 2004; Henrotin et al., 2014; Singh et al., 2015; Simental-Mendía et al., 2018). The safety of CS, on the other hand, is guaranteed. Researchers discovered that giving glucosamine hydrochloride to OA mice prevented not only the loss of GAGs and proteoglycans in articular cartilage but also bone resorption by inhibiting RANKL (Ivanovska and Dimitrova, 2011; Henrotin et al., 2012). Glucosamine hydrochloride was also demonstrated to block the production of IL-6 and to upregulate the production of IL-10 by the synovial membrane in the same study. And scholars have developed a controlled drug delivery system that co-delivers diacerein and GS, for the treatment of osteoarthritic knee. Based on the results, it can be concluded that this new formulation could induce chondroprotection with a downturn effect on inflammatory biomarkers. Despite that the effectiveness of glucosamine is controversial (Towheed et al., 2005; Bruyère et al., 2016; Roman-Blas et al., 2017; Runhaar et al., 2017; Ogata et al., 2018), glucosamine is widely considered to be safe, and no serious or fatal adverse events have ever been reported. Glucocorticoids (GCs) also alter whole-body homeostasis, modulate immunological responses and brain functions, change tissue integrity, and affect the skeletal system (Hartmann et al., 2016). The glucocorticoid receptor can interact with NF-κB and AP-1 to limit the transcription of these genes, according to researchers (Mihailidou et al., 2016). In addition, GC inhibited the expression of proinflammatory mediators TNF-α, IL-6, and MMP-9, indicating that it has anti-inflammatory properties (Zimmermann et al., 2009). Furthermore, GC can inhibit IL-1-induced collagen breakdown and suppress MMP-1 and MMP-3 production. As a result, it has the ability to protect articular cartilage, prevent ECM breakdown, enhance ECM production, and stimulate chondrocyte proliferation (Lu et al., 2011; Li et al., 2015). Long-term use of GCs, as well as disrupted negative feedback loops or persistent stress, can lead to serious consequences (McDonough et al., 2008). Insulin resistance, muscular and skin atrophies, depression, and severe skeletal consequences are among them. As a result, the use of GCs should be closely regulated. Many tissues and fluids contain hyaluronic acid (HA). The amount of HA in different joints and species varies greatly (Gupta et al., 2019). By binding to CD44, HA can preserve cartilage, because HA-CD44 inhibits IL-1β, MMP-1, MMP-2, MMP-3, MMP-9, and MMP-13 expression is reduced (Brun et al., 2012; Pohlig et al., 2016). HA has been shown in numerous studies to have anti-inflammatory properties (Litwiniuk et al., 2016; Pontes-Quero et al., 2019; How et al., 2020). IL-1 was suppressed after HA binding to CD44. Furthermore, MMPs were downregulated after IL-1β suppression, which contributed to the anti-inflammatory action of HA. The binding of HA to CD44 can block inflammatory factors such as IL-8, IL-6, and TNF-α, as well as upregulate the anti-inflammatory impact (Chang et al., 2012). Table 1 is a summary of properties of the above pharmaceutical reagents.

**TABLE 1:** The influence of chemical drugs in cytokine secretion and the underlying molecular mechanisms in OA.

Classification	Compound	Molecular formula	Cytokines	Pathway	Administration
NSAIDs	Celecoxib	C_17_H_14_F_3_N_3_O_2_S	PGs, NO↓ IL-1↓ IL-6, TNF-α↓ MMPs↓	COX, NF-κB	Oral
	Diclofenac	C_14_H_11_Cl_2_NO_2_			Oral
	Etofenamate	C_18_H_18_F_3_NO_4_			Topical
	Etoricoxib	C_18_H_15_ClN_2_O_2_S			Oral
	Indomethacin	C_19_H_16_ClNO_4_			Oral/Rectal
	Licofelone	C_23_H_22_ClNO_2_			Oral
	Naproxen	C_14_H_14_O_3_			Oral
	Nimesulide	C_13_H_12_N_2_O_5_S			Oral
	Rofecoxib	C_17_H_14_O_4_S			Oral
	Tiaprofenic acid	C_14_H_12_O_3_S			Oral
Glucocorticoids	Betamethasone	C_22_H_29_FO_5_	IL-1, IL-6↓ TNF-α↓ MMPs↓	NF-κB AP-1	Intra-articular inject
	Methylprednisolone	C_22_H_30_O_5_			
	Triamcinolone	C_21_H_27_FO_6_			
Symptomatic Slow-Acting Drugs in Osteoarthritis	Chondroitin sulfate	C_13_H_21_NO_15_S	IL-1, IL-6↓ TNF-α↓ MMPs↓ Col Ⅱ ↑ IL-6↓	NF-κB iNOS Bcl-2	Oral/*i.m.*
	Glucosamine sulfate	2C_6_H_13_NO_5_ H_2_O_4_S	TGF-β3↓ BMP-2↓ IL-10↓ Col Ⅱ ↑ PGs↓	RANKL NF-κB	Oral
Analgesics	Acetaminophen	C_8_H_9_NO_2_	IL-8, IL-6↓ PGE2↓	COX-1, COX-2	Oral
Intra-Articular Injection Medications	Hyaluronic acid	C_14_H_22_NNaO_11_	TNF-α↓ MMPs↓	HA-CD44 NF-κB	Intra-articular inject

Up to now, some of the above drugs have been carried out for clinical trials (Table 2). These drugs have the potential to treat OA. Margreet found that Lutikizumab (an IL-1 inhibitor) treatment compared with placebo was associated with significant reductions in serum IL-1α and IL-1β levels (Kloppenburg et al., 2019). What’s more, Lutikizumab can significantly decrease the levels of neutrophils, high-sensitivity C reactive protein and serum collagen type I compared with placebo and Serum collagen type were also reduced. However, despite the adequate blockade of IL-1, lutikizumab did not improve pain or imaging outcomes in erosive Hand OA compared with placebo. In another phase II multicenter double-blind study, knee OA patients received a single intra-articular injection of placebo or CNTX-4975 (an injectable form of highly purified trans-capsaicin). The results show that a single intra-articular injection of CNTX-4975 was effective in providing a significant and clinically meaningful reduction in pain that occurs while walking on a flat surface in patients with chronic moderate-to-severe OA knee pain (Stevens et al., 2019). The improvement in pain was associated with a reduction in knee stiffness and an improvement in function compared to placebo. To evaluate the efficacy, safety, and tolerability of MIV-711 in participants with symptomatic, radiographic knee osteoarthritis. Philip designed a 26-week randomized, double-blind, placebo-controlled phase 2a study (Conaghan et al., 2020). Progression of medial femoral bone area was significantly reduced in both MIV-711 treatment groups compared with placebo, and medial femoral cartilage thinning was reduced in the group receiving 100 mg/d. Significant reductions in bone resorption and cartilage degradation biomarkers were also observed. In conclusion, MIV-711 showed no beneficial effects on osteoarthritic knee pain in this study. However, statistically significant reductions in bone and cartilage osteoarthritis manifestations were observed, along with a reassuring safety profile. Marie-Pierre’s group want to explore if inhibition of inducible nitric oxide synthase (iNOS) with cindunistat hydrochloride maleate will slow the progression of osteoarthritis (Hellio le Graverand et al., 2013). Their statistics show that irreversible iNOS inhibitor Cindunistat did not reduce the rate of joint space narrowing in patients with knee OA in comparison with placebo and iNOS inhibition did not slow OA progression. What’s more, the loss of efficacy over time and lack of effect in patients suggest that alternative biochemical catabolic pathways overcame the effects of NO inhibition and/or that the consequences of the increased intra-articular stress may not have been amenable to iNOS inhibition alone. In Felix Eckstein’s clinic trial, they applied the recombinant human fibroblast growth factor 18 (sprifermin) to patients, compared with placebo (Eckstein et al., 2020). The current results support the concept that sprifermin increases cartilage thickness, and reduces cartilage loss. They showed structural modification of cartilage thickness with sprifermin. Sprifermin should be evaluated further in clinical trials as a potential DMOAD for knee OA. To explore the disease modifying effect of strontium ranelate (SrRan) treatment on cartilage volume loss and bone marrow lesions in a subset of OA patients, Pelletier conducted a phase III clinical trial (Pelletier et al., 2015). They find that treatment with SrRan 2 g/day can reduce knee OA cartilage volume loss predominantly in the plateau and that in patients with bone marrow lesions, a protective effect of SrRan was found to substantially reduce the cartilage volume loss in the medial plateau. Taken together, these findings showed that SrRan has a DMOAD effect in knee OA patients. Tanezumab, a monoclonal antibody, inhibits nerve growth factors and reduces chronic pain. Balanescu conducted a study to evaluate the efficacy and safety of tanezumab added to oral diclofenac sustained release in patients with hip or knee OA pain (Balanescu et al., 2014). The study reported that the addition of tanezumab to diclofenac sustained release resulted in significant improvements in pain, function and global assessments in patients with OA. Although no new safety signals were observed, the higher incidence of adverse events in the tanezumab + diclofenac group suggests that combination therapy is unfavorable. Further investigations of tanezumab monotherapy for OA pain treatment are required. Yazici conducted a phase II clinical trial to assess the safety and efficacy of the novel Wnt/β pathway modulator lorecivivint (SM04690) for treating pain and inhibiting structural progression in moderate-to-severe symptomatic knee OA. The results data indicated that lorecivivint have improvements in pain and function compared with placebo. This study identified a target group of subjects with unilateral symptomatic knee OA and a potentially optimal dose of LOR (0.07 mg). The clinical and radiographic outcomes warrant additional studies of the potential of LOR for both analgesia and disease-modifying activity in knee OA. Paula’s study assessed the efficacy, general safety, and joint safety of fasinumab, an anti-nerve growth factor monoclonal antibody, in OA pain (Dakin et al., 2019). In this phase IIb/III study, fasinumab demonstrated a substantial degree of analgesia in patients with moderate-to-severe pain from OA, without clear evidence of dependence on dose level for efficacy. This represents an important, previously unaddressed patient population. Fasinumab was well tolerated by most patients, with a clear dose-dependent increase in joint-related abnormalities. The observation that the efficacy of lower doses was similar to that of higher doses but was associated with lower rates of arthropathy demands that future studies explore the benefit versus risk at these lower doses of fasinumab. When lifestyle modification and drug treatment do not work, surgery is needed. Arthroscopy is a minimally invasive surgery for eliminating meniscal tears and debridement of loose articular cartilage and it is one of the most prevalent surgeries (Chang et al., 2012; Kloppenburg et al., 2019). Nonspecific treatment for OA is not suggested because of the multiple factors that influence success rates (Hellio le Graverand et al., 2013; Stevens et al., 2019; Conaghan et al., 2020; Eckstein et al., 2020). Knee replacement surgery has been performed consistently for more than four decades. Because severe arthritis of the knee usually affects only one compartment, it can be treated with either unicompartmental (UKA) or complete knee arthroplasty (TKA). UKA has a speedier recovery, fewer problems, and better function (Balanescu et al., 2014; Pelletier et al., 2015; Dakin et al., 2019). While TKA is only used as a last resort for patients with OA. TKA is typically advised for older patients who have terrible knee pain, unacceptable activity limitations that result in the loss of important activities, and severe end-stage OA of the joint (Song et al., 2018).

**TABLE 2:** Clinical trials of some chemical drugs for OA.

Classification	Drug	Therapeutic influence	Administration	Clinical trial number
Anti-IL-1	Lutikizumab (ABt-981)	Downregulate IL-1α and IL-1β levels, decreased the levels of neutrophils, hsCRP and serum C1M	Subcutaneous injection	NCT02384538
				Phase IIa
Trans-Capsaicin	CNTX- 4975	Alleviate pain	Intra-articular injection	NCT02558439
				Phase II
Cathepsin K Inhibitor	MIV-710	Reductions in bone and cartilage osteoarthritis manifestations	Oral	NCT03037489
				Phase IIa
Selective iNOS Inhibitor	Cindunistat (SD-6010)	Inhibition of iNOS and its downstream products	Oral	NCT00565812
				Phase III
Recombinant human fibroblast growth factor 18	Sprifermin	Increases cartilage thickness, and reduces cartilage loss	Intra-articular injection	NCT01919164
				Phase II
Antiosteoporotic agent	Strontium ranelate (SrRan)	Downregulate MMPs and NF-κB, activate osteoprotegerin	Oral	NCT03937518
				Phase III
Nerve growth factor inhibition	Tanezumab	Downregulate Nerve growth factor level	Intravenous injection	NCT00864097
				Phase III
	Fasinumab		Subcutaneous injection	NCT02447276
				Phase IIb/III
	Fulranumab		Subcutaneous injection	NCT00973141
				Phase II
Wnt pathway modulator	Lorecivivint (SM04690)	Inhibit CDC-like kinase 2 and dual-specificity tyrosine phosphorylation-regulated kinase 1A, affect Wnt pathway	Intra-articular injection	NCT02536833
				Phase IIa

Table 2 is a summary of properties of the above pharmaceutical reagents.

## Active ingredients of medicinal plants for osteoarthritis

In clinic, antipyretic analgesics, NSAIDs, opioids, and GCs are frequently used to treat OA. However, these medications are just symptomatic and have several negative effects. An increased risk of gastrointestinal bleeding and a slight rise in systolic blood pressure are two adverse effects for which there is solid evidence (Evans et al., 2014; Maudens et al., 2018; Zhang et al., 2020b). Insulin resistance, muscular and skin atrophies, depression, and severe skeletal consequences are all examples of GCs. As a result, developing a new drug candidate and/or improving the drug delivery safety and efficacy is critical for the treatment of OA. Traditional Chinese medicine has long been used to treat a variety of ailments, and it has the benefits of being affordable, widely available, and have fewer adverse effects (Song et al., 2018). Scholars looked into the treatment potential of traditional Chinese medications for OA, and the findings revealed that they are both effective and safe (Liu et al., 2013; Chen et al., 2017).

Matrine is an alkaloid obtained from the traditional Chinese medicine *Sophora flavescens* Aiton. Basic research on matrine’s anti-inflammatory properties is currently in substantial volume, indicating that matrine possesses pharmacological activity and therapeutic application potential (Zhang et al., 2020a). Matrine inhibits MAPK and NF-κB activation in human chondrocytes *in vitro* and reduces IL-1β-induced MMP synthesis, protecting chondrocytes against extracellular matrix degradation by MMPs (Lu et al., 2015).

Sinomenine is a bioactive alkaloid produced from the Chinese medicinal plant *Sabia japonica* Maxim. Sinomenine reduces the number of CD11bF4/80CD64 synovial macrophages and CD11bLy6CCD43 monocytes/macrophages in CIA mice. In CIA mice, a decrease in the quantity of these macrophages suppresses the release of inflammatory cytokines. Sinomenine can thereby control the inflammatory response by inhibiting the activities of IL-1α, IL-1β, TNF-α, and IL-6 (Liu et al., 2018; Gao et al., 2019).

Osthole is a natural coumarin derived from the *Cnidium monnieri* (L.) Cuss that is extensively used in Traditional Chinese Medicine clinical practice. Recent research has discovered that osthole possesses antioxidant, anticancer, anti-inflammatory, and immunomodulatory properties (Wang et al., 2007; You et al., 2009; Zhang et al., 2015). Excessive stimulation of the PI3K/Akt pathway has been demonstrated to increase MMP expression in previous investigations (Katsara et al., 2017). Inflammation, immunological response, and apoptosis are all regulated by the NF-κB pathway. Researchers have discovered that osthole can drastically lower critical proteins in the PI3K/Akt/NF-κB pathway, hence alleviating OA. Toll-like receptor 4 (TLR4) activation is linked to chondrocyte inflammation and catabolism, according to researchers (Yan et al., 2019). TLR4 and its downstream NF-κB are inhibited by curcumin, which reduces synovial inflammation.

Curcumin, extract from *Curcuma longa* L. can also protect chondrocytes and maintain cartilage homeostasis by activating the ERK1/2 pathway and inhibiting the Akt/mTOR pathway (Zhang et al., 2018). Curcumin’s anti-OA properties can possibly be attributed to other factors. Curcumin, for example, can decrease chondrocyte death and enhance chondrocyte proliferation by mediating the Wnt/*β*-Catenin pathway (Wang et al., 2017). Inhibiting NF-κB signal transduction can also diminish chondrocyte extracellular matrix breakdown.

Loganin is a prominent iridoid glycoside and one of *Strychnos nux-vomica* L.’ quality control indicators. Loganin can boost Col2a1 expression while lowering MMP-3, MMP-13, Col10, cryopyrin, and caspase-1 expression in cartilage (Hu et al., 2020). In subchondral bone, loganin reduced the expression of CD31 and endomucin. Furthermore, loganin inhibited p65 protein nuclear translocation and decreased the quantity of p-IκB in chondrocytes (Yang et al., 2019). According to these findings, Loganin suppresses NF-κB signaling and reduces cartilage matrix degradation and chondrocyte pyroptosis in articular cartilage.

Morroniside is a significant iridoid glycoside and one of *Cornus officinalis* Sieb.et Zucc.’ quality control criteria. Morroniside promotes cartilage matrix formation by increasing collagen type II expression and reducing chondrocyte pyroptosis. Morroniside decreased the synthesis of MMP-13, caspase-1, and nod-like receptor protein-3 (NLRP3) in IL-1β-stimulated chondrocytes (Park et al., 2021). Morroniside also slowed the course of OA by increasing chondrocyte proliferation and decreasing chondrocyte death. Morroniside has been shown to block NF-κB signaling, slowing the development of OA (Yu et al., 2021).

Madecassoside is a triterpenoid component obtained from the Gotu kola plant [*Centella asiatica* (L.) Urban] that has antioxidative and anti-inflammatory activities. Madecassoside has been demonstrated to successfully suppress IL-1β-induced overexpression of MMP-3, MMP-13, inducible nitric oxide synthase (iNOS), and COX-2, as well as type II collagen and sox9 degradation (Moqbel et al., 2020). Madecassoside was also able to inhibit IL-1β-induced phosphorylation of p65 in osteoarthritic chondrocytes. Furthermore, as compared to the OA group, madecassoside reduced the OARSI score and avoided cartilage deterioration in a rat OA model. These findings demonstrated that madecassoside inhibits the NF-κB signaling pathway, lowering IL-1β-induced chondrocyte inflammation, implying that MA might be used to treat OA patients.

Quercitrin is a yellow-colored flavonoid that is a substantial component of *Taxillus sutchuenensis* (Lecomte) Danser’ total flavonoids. By stimulating the p110/AKT/mTOR signaling pathway, quercitrin can decrease MMP-13 production and enhance collagen II accumulation, promoting cell proliferation and delaying ECM breakdown (Guo et al., 2021). According to the research, quercitrin targets p110 to produce its anti-OA properties. Grape seed extract contains resveratrol, which is a polyphenol phytoalexin (Xu et al., 2019). Resveratrol can block the degradation of IκB-α and the activation of NF-κB caused by IL-1β.

Resveratrol can significantly reduce the inflammatory response induced by IL-1β in OA chondrocytes, including MMP-13, MMP-3, and MMP-1. Resveratrol can also boost collagen-II and aggrecan levels. As a result, Resveratrol holds a lot of promise in the treatment of OA. Anti-inflammatory and antioxidant effects have been discovered in fraxetin. It is a coumarin chemical isolated from *Fraxini Cortex* that is frequently utilized and investigated. According to the findings, fraxetin suppressed inflammatory mediator release and prevented chondrocyte death produced by IL-1β through modulating the TLR4/myeloid differentiation primary response 88/NF-κB pathway in chondrocytes (Wang et al., 2020). In addition, fraxetin inhibited MMP-13 overexpression and collagen II breakdown in the ECM. The findings revealed that fraxetin might prevent cartilage from deterioration.

Ligustilide, a key ingredient in the plant *Angelicae Sinensis Radix*, has anti-inflammatory properties. Ligustilide inhibits NF-κB activation *via* the PI3K/AKT pathway, reducing IL-1β-induced chondrocyte inflammation and ECM breakdown (Li et al., 2018). In human OA chondrocytes, Ligustilide inhibits the production of PGE2, iNOS, COX-2, MMP-3, MMP-13, and ADAMTS-5, as well as the breakdown of aggrecan and collagen II. Ligustilide has the potential to be an effective OA treatment.

Icariin, an *Epimedium* extract, has been shown to have anti-osteoporotic and anti-inflammatory properties in OA patients. Icariin might reduce pyroptosis by suppressing the NLRP3 signaling-mediated caspase-1 pathway, which is important in the pathogenesis of OA, to reduce chondrocyte damage and the incidence of OA. Icariin may be a promising target drug for the treatment of OA. Icariin appears to be a viable target drug for treating OA.

Baicalein, which is derived from the *Scutellaria baicalensis* Georgi, is commonly used in anti-inflammatory treatments. Baicalein might successfully relieve OA by improving chondrocyte apoptotic and catabolic phenotypes and subchondral bone remodeling (Chen et al., 2015). Baicalein was discovered to limit preosteoblast differentiation and proliferation while triggering preosteoblast death, hence controlling subchondral bone remodeling (Li et al., 2021). Baicalein can also reduce vascularisation and synovial cell proliferation, helping to regulate synovitis and ease subchondral bone sclerosis. Baicalein may be an effective treatment for OA since it regulates many targets.

modin is a natural anthraquinone isolated from the root and rhizome of *Rheum palmatum* L. that has previously been demonstrated to have antibacterial (Kim et al., 2005), anti-cancer (Kaneshiro et al., 2006; Lin et al., 2010) and anti-inflammatory (Alisi et al., 2012) activities. Emodin may have anti-osteoarthritic actions *via* suppressing the NF-κB and Wnt/β-catenin pathways, which reduce the production of MMP-3, MMP-13, ADAMTS-4, and ADAMTS-5 (161). Cell proliferation, migration, and differentiation, as well as cartilage homeostasis and joint remodeling, are all influenced by the Wnt/*β*-Catenin pathway. Emodin has the potential to cure OA, according to the findings.

The active chemical found in medicinal plants holds a lot of promise for treating OA. However, further clinical trials are needed to validate their usefulness and safety.

Table 3 shows the therapeutic influence of the above natural products for OA.

**TABLE 3:** The therapeutic influence of natural products in OA.

Compound	Molecular formula	Cytokines	Pathway
Matrine	C_15_H_24_N_2_O	IL-1, IL-6, TNF-α, MMPs↓ Col Ⅱ↑	NF-κB, MAPK
Sinomenine	C_19_H_23_NO_4_	MMPs, IL-1α, IL-1β↓ TNF-α, IL-6↓	NF-κB
Osthole	C_15_H_16_O_3_	IL-1, IL-6, TNF-α, MMPs↓	PI3K/Akt/NF-κB
Puerarin	C_21_H_20_O_9_	IL-1, IL-6, TNF-α, MMPs↓ Col Ⅱ↑	NF-κB
Ginsenoside Rg1	C_42_H_72_O_14_	MMP-3, IL-1, TNF-α↓ Col Ⅱ↑	NF-κB
Curcumin	C_21_H_20_O_6_	IL-1, IL-6, TNF-α↓	JAK2/STAT3, NF-κB, Akt/mTOR
Loganin	C_17_H_26_O_10_	MMPs↓ Col Ⅱ↑	NF/κB, PI3K/Akt
Morroniside	C_17_H_26_O_11_	MMP-1, MMP-13↓ Col Ⅱ↑	NF-κB
Madecassoside	C_30_H_48_O_6_	MMP-13, COX-2↓ Col Ⅱ↑	p65/NF-κB
Quercitrin	C_21_H_20_O_11_	MMP-13↓ Col Ⅱ↑	p110α/AKT/mTOR
Resveratrol	C_14_H_12_O_3_	IL-1, IL-6, TNF-α, MMPs↓ COX-2↓	NF-κB
Andrographolide	C_20_H_30_O_5_	IL-1, IL-6, TNF-α↓	NF-κB
Fraxetin	C_10_H_8_O_5_	IL-1, IL-6, TNF-α, MMP-13↓ Col Ⅱ↑	TLR4/My D88/NF-κB
Ligustilide	C_12_H_14_O_2_	PGE2, iNOS, COX-2↓ MMP-13, ADAMTS-5↓	PI3K/Akt/NF-κB
Icariin	C_33_H_40_O_15_	NLRP3, IL-1β, IL-18↓ MMP-1, MMP-13↓ Col Ⅱ↑	p38, ERK, JNK, Wnt/*β*-Catenin
Baicalein	C_15_H_10_O_5_	MMPs↓	MAPK
Emodin	C_15_H_10_O_5_	MMP-3, MMP-13↓ ADAMTS-4↓	NF-κB, Wnt/*β*-Catenin

### Delivery strategies

As a topical disease, innovative delivery options such as intra-articular injection or transdermal delivery have been extensively researched in addition to the traditional oral and systemic administration routes. Many biomaterials have been investigated for the design of novel drug delivery systems for the treatment of OA to improve the efficacy of the delivery vehicles.

Oral delivery is still the most common approach in drug administration for OA (Sastry et al., 2000; Sant et al., 2012). However, oral distribution is hampered by physiological hurdles such as the harsh gastrointestinal environment, as well as enzymatic degradation and tolerance, which make it difficult to use oral delivery methods in clinical settings (Homayun et al., 2019). For example, the most popular and traditional therapy for OA pain relief is NSAIDs. However, NSAIDs have a number of serious adverse effects, including toxicity and a severe gastrointestinal response (Meng and Li, 2017; Polderman et al., 2019). Improved oral administration such as by the nano or micro formulations can enhance medication absorption with long effects and lessen gastrointestinal side effects and frequent administrations. For example, HA-Liposomal (Lipo)-DIC has been shown to achieve an effective working concentration in 4 h and maintain it for at least 168 h (Chang et al., 2021).

### Intra-articular injection

As a localized disease, OA may be treated through intra-articular (IA) injections (Gerwin et al., 2006). Intra-articular drug delivery offers a variety of advantages, including higher local bioavailability, less systemic exposure, fewer side effect, and lower cost (Evans et al., 2014; Emami et al., 2018). However, because of the dense avascular cartilage matrix made up of negatively charged glycosaminoglycans (GAGs), medicines injected locally will quickly leave the joint region and disperse and travel slowly. The need for biomaterial-based drug delivery vehicles that can increase medication bioavailability in target tissues notwithstanding the challenges (Maudens et al., 2018). IA injectable formulations of glucocorticoids and hyaluronic acid are available (Zhang et al., 2020b). And the majority of them are in the form of solutions or suspensions. Due to the fast disappearance of classic injectable formulations in joints, new drug delivery methods are expected to be developed yet.

Microspheres can not only protect pharmaceuticals *in vivo* from numerous causes but also slow down their release. It reduces delivery durations and doses while avoiding the harmful side effects of systemic absorption, providing essential theoretical support for OA research and therapy. Triamcinolone acetonide (TAA), a traditional corticosteroid, relieves synovitis and discomfort, but only temporarily. Local release of TAA based on biomaterials may extend pain relief without the need for numerous injections (Burt et al., 2009).

**FIGURE 3:**
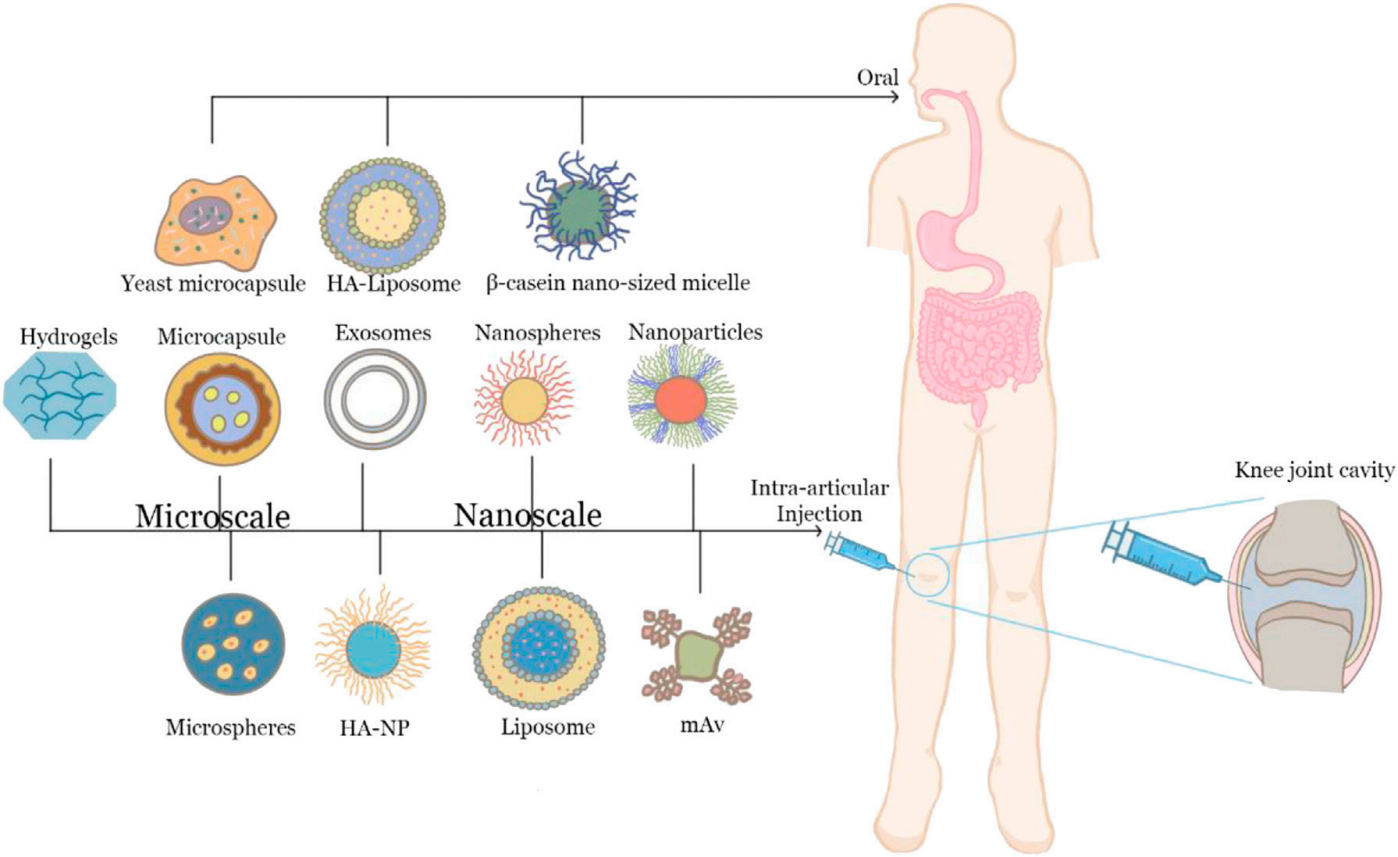
Oral delivery and intra-articular injection systems for OA.

Scholars discovered that hyaluronic acid nanoparticle/hydrogel (HA-NP) exhibited resistance to hyaluronidase digestion *in vitro* and long-term retention ability in the knee joint *in vivo*. The injectable hydrogel-based drug delivery system-dexamethasone hydrogels have a consistent release profile from the start, with a content of 22 percent of the original medication released in 5 days. Furthermore, these drug delivery methods are less cytotoxic and support good cell viability in chondrocytes and osteoblast cells. Intra-articular injection can reduce gastrointestinal reactions, avoid the first-pass effect, and ensure that drugs can be delivered to the articular cavity. However, intra-articular injection needs to puncture the skin, which is invasive and may cause inflammation and bring secondary damage to OA patients. What’s more, 50% of the clinical intra-articular injections will be injected outside the joint cavity. Therefore, it is still necessary to develop new delivery systems to prolong the drug retention time in the joint and reduce the frequency of drug delivery. Above all, Figure 3 have shown oral and intra-articular delivery systems for OA.

### Transdermal delivery

OA is a localized disease that often occurs in the knee, hand and hip joints. Therefore, skin penetration-based topical administration may commonly meet the needs of OA treatment. Transdermal drug administration can allow the medication to enter circulation at a steady pace and continuously *via* the skin. Compared to the oral route or intra-articular injection, transdermal administration offers a number of benefits (Prausnitz and Langer, 2008). Transdermal administration can minimize administration frequency, improve slow-release effectiveness, and improve patient compliance (Yin and Smith, 2016; Brown and Williams, 2019). However, the stratum corneum, which functions as the first protective layer of the skin and limits medication absorption, poses the biggest obstacle in drug administration *via* the transdermal method (Hadgraft and Lane, 2005). To enhance the skin penetration of drugs, various physical and chemical means of promoting permeation have been attempted for transdermal delivery of OA drugs.

Numerous chemical enhancers are capable of disrupting the highly ordered bilayer structures of intracellular lipids found in the stratum corneum by inserting amphiphilic molecules into these bilayers to disrupt molecular packing or by extracting lipids using solvents and surfactants to create nanometer-scale lipid packing defects (Prausnitz and Langer, 2008). However, the most significant limitation of this technique is that skin irritation may induce allergic responses in individuals. Iontophoresis is another technique for improving transdermal penetration by supplying an electrical driving force. Due to the fact that iontophoresis does not affect the epidermal barrier directly, it is often applied to tiny charged molecules and certain macromolecules up to a few thousand Daltons (Kalia et al., 2004).

Cavitational ultrasound that uses the bubbles to oscillate and collapse at the skin surface to generate localized shock waves and liquid microjets directed at the stratum corneum induces the disruption in the lipid structure of the stratum corneum and can increase skin permeability for many hours. Additionally, thermal effects from ultrasound have been suggested to impact sonophoretic skin contact favorably by increasing the drug diffusion and the skin permeability coefficients (Canavese et al., 2018; Park et al., 2016).

Articular thermotherapy induces vasodilation and increases blood flow around the joints, thereby reducing pain and joint stiffness (Choi et al., 2015). Besides this, heaters can also help drugs cross the skin. When the thermos therapy is applied, solubility and diffusivity of inorganic and organic drugs increase from the drug-containing layer (Bagherifard et al., 2016). Transdermal drug permeation is increased by the application of heat, which enhances the circulation of bodily fluid, the permeability of blood vessel walls, and the rate-limiting membrane permeability (Lee et al., 2018).

**FIGURE 4:**
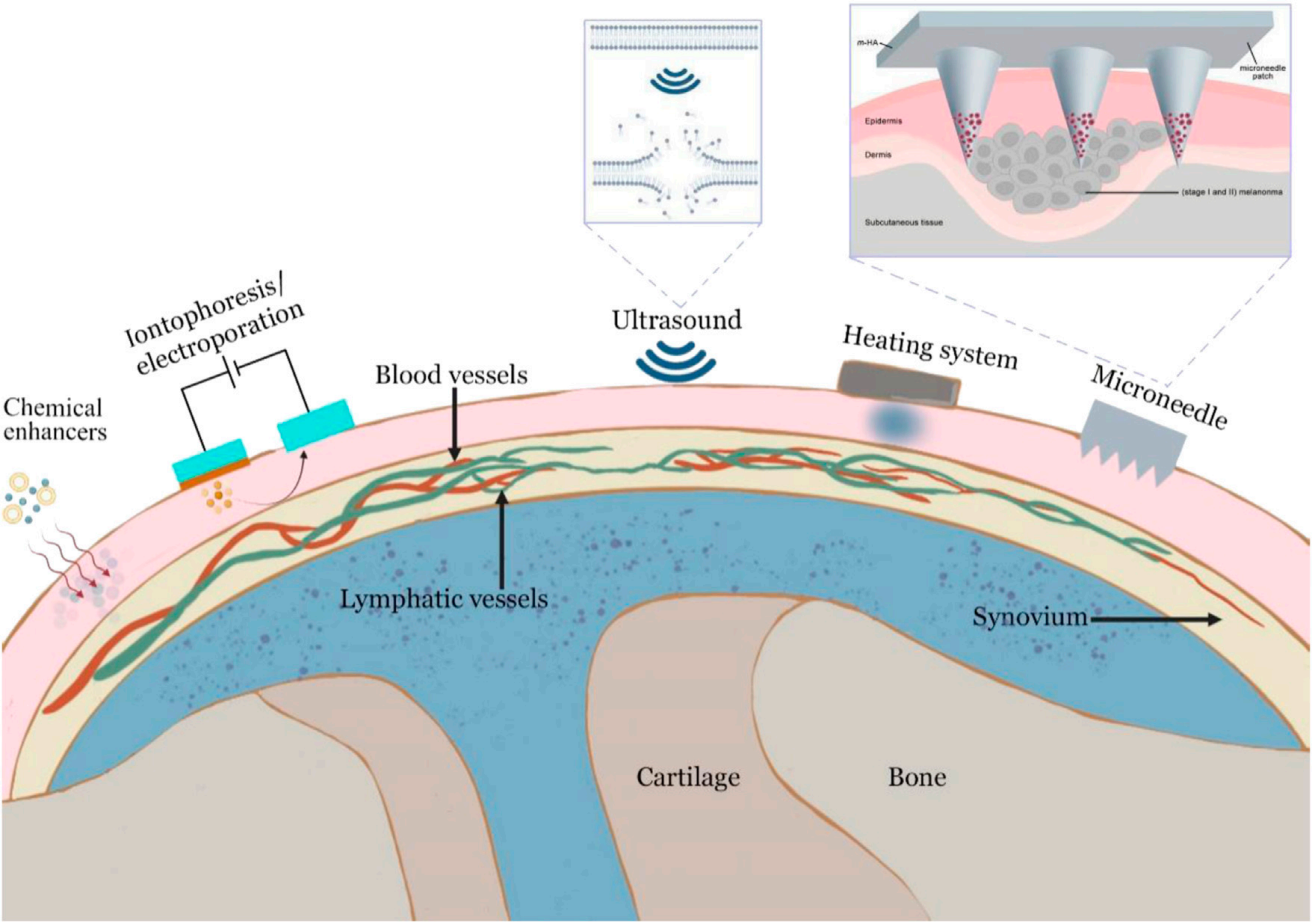
Skin penetration enhancement strategies.

Microneedles are tiny and cheap needles that can carry medications through the skin in the least invasive way (HS et al., 2014; Kennedy et al., 2017). Furthermore, microneedles allow for the regulated and sustained release of medications based on the demands of the patients. Microneedles, in general, could increase skin permeability by creating micron-scale pathways into the skin, and actively drive drugs into the skin either as coated or encapsulated cargo introduced during microneedle insertion or *via* convective flow through hollow microneedles. Skin penetration enhancement strategies are shown in Figure 4. And we have summarized the advantages and disadvantages between different delivery systems in Table 4.

**TABLE 4:** Advantages and disadvantages between different delivery systems.

Administration	Delivery vehicles	Delivered drug	Disadvantages	Advantages
Oral	Microcapsules/Yeast	IL-1β shRNA	Gastrointestinal reaction, high manufacturing cost	Enhance medication absorption, lessen gastrointestinal side effects, lower toxicity, increase bioavailability, good patient compliance
	Liposomes/DOPC, HA	Diclofenac		
Intra-articular injection	Microspheres/PEA	Triamcinolone acetonide	Risk of infection, cause patient pain, poor patient compliance, instability, safety of biomaterials	Enhance bioavailability and effectiveness, reducing the injury rate of intra-articular glucocorticoid delivery and the side effects of glucocorticoid drugs. Delay drug release
	Nanospheres/Sodium hyaluronate	HA		
Transdermal delivery	Liposomes/Microneedles Liposomes/Ultrasound Liposomes/Thermos therapy	Triptolide	Risk of infection, low drug loading content, allergic responses	Delay drug release, minimize administration frequency, improved drug penetration, reduce joint damage, improve patient compliance
		Diclofenac		
		Fentanyl		

## Prospect

OA is a chronic joint disease characterized by degeneration of articular cartilage and hyperosteogeny around the joint. At present, the molecular mechanism of OA is still not clear. The process of modern science and medicine will help us to know better about the pathogenesis of OA and thus more therapeutic targets can be excavated in follow-up work (Rahmati et al., 2017). Lifestyle modification is still the most effective way in the treatment of OA. Doing more exercise to lose weight and keep fit’s not only economical but also can control or slow down the course of OA and reduce the mortality rate of OA. Acetaminophen and NSAIDs are often used in the clinical treatment of OA, but long-term use of these drugs will lead to nephrotoxicity and gastrointestinal reactions. For this reason, it is urgent to develop new drugs for treating OA. Traditional Chinese medicine accumulated a wealth of experience in the treatment of osteoarthritis. At present, many studies that have been focused on the therapeutic effects of active ingredients from medicinal plants, such as ginsenoside, matrine and sinomenine. They were shown to inhibit the expression of pro-inflammatory factors such as IL-1 and TNF-α in diseased tissues, and the downregulation of IL-6 can alleviate the local inflammatory response. Meanwhile, they can also reduce the expression of matrix metalloproteinases to inhibit the interpretation of extracellular matrix of chondrocytes and protect cartilage and according to the current studies, these active ingredients did not show strong cytotoxicity. There is an extensive prospect to explore the application of traditional Chinese medicine in OA.

Another challenge for pharmaceutical treatment in OA lies in the design of cartilage-targeting drug delivery systems. Oral administration frequently leads to fierce gastrointestinal reactions and low bioavailability because of the first-pass effect. Intra-articular injection is an invasive method to the joints. Therefore, various DDSs such as nanoparticles, liposomes and hydrogels based on transdermal/topical delivery systems have caused increasing attention to reducing the frequency of intra-articular injections, increasing patient compliance, and reducing the risks along with the conventional preparations.

Furthermore, as a big challenge to deliver drugs into chondrocytes, we can use physical technologies such as ultrasound and iontophoresis to assist the process of drug delivery and build intelligent local drug delivery systems which help researchers to provide new strategies for the treatment of OA. Besides, new approaches that simultaneously deliver chemical drugs and biological drugs aiming to enhance drug efficacy and reduce the side effects might be also combined to develop the next generation therapeutic system for OA treatment. Because the combination of several different types of drugs may work synergistically, the advanced vehicles that can co-deliver multiple drugs or release them in a responsive manner are highly proposed. However, it presents several new challenges that must be dealt with and a thorough biological assessment is needed for determining the drug combination. Another important aspect is the determination of the best mass ratio of each agent within a co-deliver drugs system which needs a systematic study to investigate the effects of different drug ratios on biological efficacy (Karsdal et al., 2016; Lotz and Caramés, 2011; Hunter, 2011; Vos et al., 2012).
